# A Weyl Fermion semimetal with surface Fermi arcs in the transition metal monopnictide TaAs class

**DOI:** 10.1038/ncomms8373

**Published:** 2015-06-12

**Authors:** Shin-Ming Huang, Su-Yang Xu, Ilya Belopolski, Chi-Cheng Lee, Guoqing Chang, BaoKai Wang, Nasser Alidoust, Guang Bian, Madhab Neupane, Chenglong Zhang, Shuang Jia, Arun Bansil, Hsin Lin, M. Zahid Hasan

**Affiliations:** 1 Centre for Advanced 2D Materials and Graphene Research Centre, National University of Singapore, 6 Science Drive 2, Singapore 117546, Singapore; 2 Department of Physics, National University of Singapore, 2 Science Drive 3, Singapore 117542, Singapore; 3 Joseph Henry Laboratory, Department of Physics, Princeton University, Princeton, New Jersey 08544, USA; 4 Princeton Center for Complex Materials, Princeton University, Princeton, New Jersey 08544, USA; 5 Department of Physics, Northeastern University, Boston, Massachusetts 02115, USA; 6 Condensed Matter and Magnet Science Group, Los Alamos National Laboratory, Los Alamos, New Mexico 87545, USA; 7 ICQM, School of Physics, Peking University, Beijing 100871, China; 8 Collaborative Innovation Center of Quantum Matter, Beijing 100871, China; 9 Princeton Center for Complex Materials, Princeton University, Princeton, New Jersey 08544, USA

## Abstract

Weyl fermions are massless chiral fermions that play an important role in quantum field theory but have never been observed as fundamental particles. A Weyl semimetal is an unusual crystal that hosts Weyl fermions as quasiparticle excitations and features Fermi arcs on its surface. Such a semimetal not only provides a condensed matter realization of the anomalies in quantum field theories but also demonstrates the topological classification beyond the gapped topological insulators. Here, we identify a topological Weyl semimetal state in the transition metal monopnictide materials class. Our first-principles calculations on TaAs reveal its bulk Weyl fermion cones and surface Fermi arcs. Our results show that in the TaAs-type materials the Weyl semimetal state does not depend on fine-tuning of chemical composition or magnetic order, which opens the door for the experimental realization of Weyl semimetals and Fermi arc surface states in real materials.

## Abstract (web summary)

Proposals for the realization of Weyl semimetals, topologically non-trivial materials which host Weyl fermion quasiparticles, have faced demanding experimental requirements. Here, the authors predict such a state in stoichiometric TaAs, arising due to the breaking of inversion symmetry.

## Graphical abstract

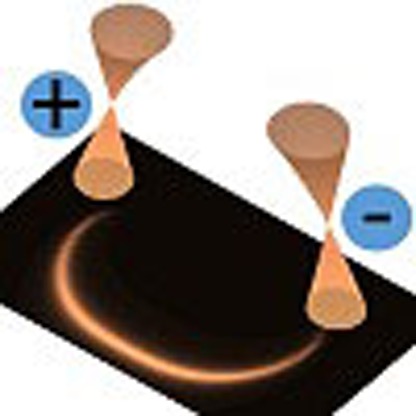

The rich correspondence between high energy and condensed matter physics has led to a deeper understanding of spontaneous symmetry breaking, phase transitions, renormalization and many other fundamental phenomena in nature, with important consequences for practical applications using magnets, superconductors and topological materials^1^,^2^,^3^,^4^. Recently, there has been considerable progress in realizing particles previously considered in high energy physics as emergent low-energy excitations of crystalline solids^4^,^5^,^6^,^7^,^8^,^9^,^10^,^11^,^12^,^13^. Materials that host these exotic particles exhibit unique properties and hold promise for applications such as topological qubits, low-power electronics and spintronics. Weyl fermions were originally considered in massless quantum electrodynamics, but have not been observed as a fundamental particle in nature^1^. Recently, it was theoretically understood that Weyl fermions can arise in some novel semimetals with non-trivial topology^5^,^6^,^7^,^12^,^13^,^14^. A Weyl semimetal has an electron band structure with singly degenerate bands that have bulk band crossings, Weyl points, with a linear dispersion relation in all three momentum space directions moving away from the Weyl point. These materials can be viewed as an exotic spin-polarized, three-dimensional (3D) version of ‘graphene' that host topological Fermi arcs. However, unlike the two-dimensional (2D) Dirac cones in graphene^9^, the 3D Dirac cones in spin–orbit materials^15^,^16^ or the 2D Dirac cone surface states of Bi_2_Se_3_(refs 10, 11), the degeneracy associated with a Weyl point depends only on the translation symmetry of the crystal lattice. This makes the unique properties associated with this electron band structure more robust. Moreover, due to its non-trivial topology, a Weyl semimetal can exhibit novel Fermi arc surface states. Both the Weyl fermions in the bulk and the Fermi arc states on the surface of a Weyl semimetal are predicted to show unusual transport phenomena. Weyl fermions in the bulk can give rise to negative magnetoresistance, the anomalous Hall effect, non-local transport and local non-conservation of ordinary current^17^,^18^,^19^,^20^. Fermi arc states on the surface are predicted to show novel quantum oscillations in magnetotransport and quantum interference effects in tunnelling spectroscopy^21^,^22^,^23^. Because of the fundamental and practical interest in Weyl semimetals, it is crucial that robust candidate materials be identified.

It is theoretically known that a Weyl semimetal can only arise in a crystal where time-reversal symmetry or inversion symmetry is broken. A number of magnetically ordered or inversion symmetry-breaking materials have been proposed as Weyl semimetal candidates^6^,^24^,^25^,^26^,^27^. However, despite extensive effort in experiments for many years, a Weyl semimetal has yet to be realized in any of the compounds predicted thus far. One major concern is that the existing proposals require either magnetic ordering in sufficiently large domains^6^,^24^,^25^,^26^ or fine-tuning of the chemical composition to within 5% in an alloy^24^,^26^,^27^, which are highly demanding in real experiments. We propose the first Weyl semimetal in a stoichiometric, inversion-breaking, single-crystal material, TaAs as a representative of the transition metal monopnictide or the TX family where T=Ta/Nb and X=As/P. Unlike previous predictions, our proposal does not depend on magnetic ordering over sufficiently large domains, because our material relies on inversion symmetry breaking rather than time-reversal symmetry breaking. The compound we propose is also stoichiometric and does not depend on fine-tuning chemical composition in an alloy as in our previous prediction^12^. We have grown single crystals of TaAs where the crystal structure is consistent with previous reports^28^. We believe that this material is the platform for the experimental realization of the long-sought-for Weyl fermion semimetal phase and Fermi arc surface states.

## Results

### Crystal and electronic structure

**Figure 1:**
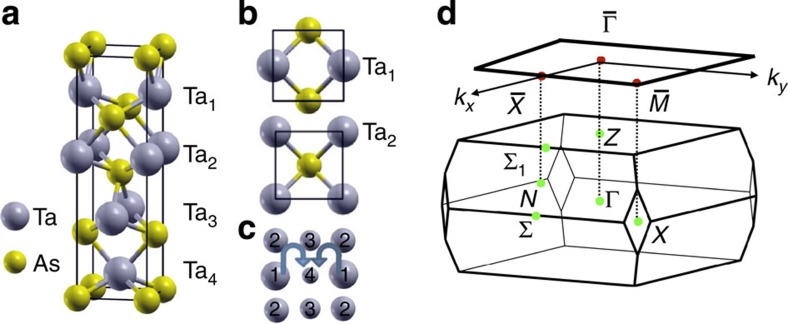
Crystal structure and Brillouin zone of the Weyl semimetal TaAs. (**a**) Body-centred tetragonal structure of TaAs, shown as stacked TaAs layers. An electric polarization is induced due to dimples in the TaAs lattice. (**b**) Shows top–down views at different vertical positions, emphasizing the fact that the crystal structure is composed of square lattices that are shifted with respect to one another. The arrangement of Ta atoms for each layer (Ta_1_ to Ta_4_) is illustrated in (**c**). (**d**) High symmetry points are noted as green and red dots in the bulk and the (001) surface Brillouin zones (BZ), respectively.

In order to find a Weyl semimetal that respects time-reversal symmetry, one needs to search for materials that break space inversion symmetry. In addition, since the Weyl nodes in a Weyl semimetal are usually not located along any high-symmetry direction, one has to calculate the band structure throughout the bulk Brillouin zone (BZ) in order to check whether there are Weyl nodes in a material or not. We have therefore searched through hundreds of inversion symmetry-breaking materials, identified the ones that are likely to be semimetal or narrow bandgap semiconductors, and then performed systematic calculations of the bulk band structures to theoretically understand the nature of their electronic and topological groundstate. Tantalum arsenide crystalizes in a body-centered tetragonal lattice system (Fig. 1a–c). The lattice constants are *a*=3.437 Å and *c*=11.656 Å, and the space group is *I*4_1_*md* (#109, *C*_4*v*_). This crystal consists of interpenetrating Ta and As sub-lattices, where the two sub-lattices are shifted by 
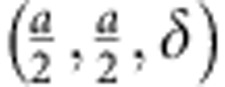
, 
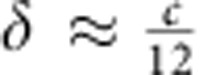
. There are two Ta atoms and two As atoms in each primitive unit cell. It is important to note that the system lacks a horizontal mirror plane and thus inversion symmetry. This makes it possible to realize an inversion breaking Weyl semimetal in TaAs. We also note that the *C*_4_ rotational symmetry is broken at the (001) surface because the system is only invariant under a four-fold rotation with a translation along the out-of-plane direction. The bulk and (001) surface BZs are shown in Fig. 1d, where high-symmetry points are also noted.

**Figure 2:**
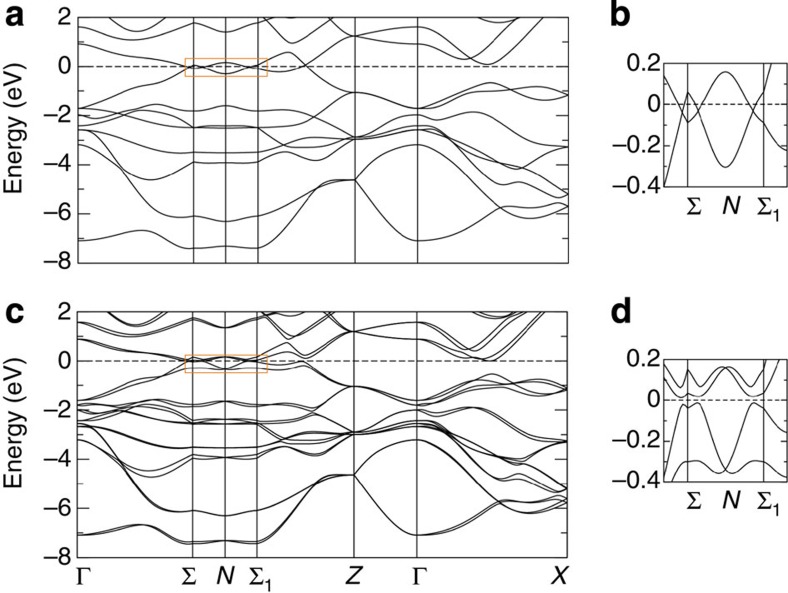
The electronic structure of the Weyl semimetal TaAs. (**a**) The bulk electronic structure of TaAs in the absence of spin–orbit coupling from DFT. (**b**) The band structure in the vicinity of the Fermi level along the Σ−*N*−Σ_1_ direction. (**c**,**d**) The same as panels **a** and **b** but in the presence of spin–orbit coupling.

The ionic model would suggest that the Ta and As atoms are in the 3^+^ and 3^−^ valence states, respectively. This indicates that the lowest valence band arises from 4*p*^6^ electrons in As and 5*d*^2^ electrons in Ta, whereas the lowest conduction band primarily consists of 5*d* electrons in Ta. However, we may expect Ta 5*d* electrons to have a broad bandwidth because of the wide extent of the atomic orbitals. This leads to strong hybridization with the As 4*p* states, which may suggest that the conduction and valence bands are not entirely separated in energy and have a small overlap, giving rise to a semimetal. In Fig. 2a, we present the bulk band structure in the absence of spin–orbit coupling. The conduction and valence bands cross each other along the Σ–*N*–Σ_1_ trajectory, which further indicates that TaAs is a semimetal. In the presence of spin–orbit coupling, the band structure is fully gapped along the high-symmetry directions considered in Fig. 2c. However, Weyl points which are shifted away from the high-symmetry lines arise after spin–orbit coupling is taken into account. Below, we consider the Weyl points in the bulk BZ. We also note that the double degeneracy of bands is lifted in the presence of spin–orbit coupling except at the Kramers' points, which confirms that TaAs breaks inversion symmetry but respects time-reversal symmetry.

### Weyl fermions form the bulk Weyl cones

**Figure 3:**
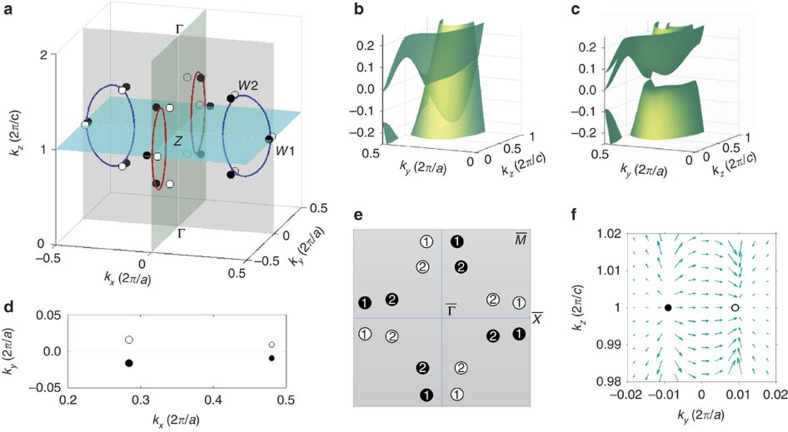
Weyl points and topological chiral charges in TaAs. (**a**) In the absence of spin–orbit coupling, there are two line nodes on the *k*_*x*_=0 mirror plane, *M*_*x*_, and two line nodes on the *k*_*y*_=0 mirror plane, *M*_*y*_. In the presence of spin–orbit coupling, each line node vaporizes into six Weyl points. The Weyl points are denoted by small circles. Black and white show the opposite chiral charges of the Weyl points. (**b**) The band structure around one of the red line nodes in the absence of spin–orbit coupling. (**c**) The band structure near one of the Weyl points at *k*_*x*_*a*=0.01*π* in the presence of spin–orbit coupling. The Weyl point is shifted away from the *k*_*x*_=0 mirror plane. (**d**) The projection of the bulk Weyl points around the 
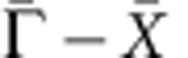
 line. (**e**) A schematic for the projection of all the Weyl points on the (001) surface Brillouin zone. We denote the eight Weyl points that are located on the *k*_*z*_=2*π*/*c* plane as *W*_1_ (1) and the other sixteen Weyl points as *W*_2_ (2). (**f**) Calculated spin texture (*S*_*y*_,*S*_*z*_) in the vicinity of two Weyl points with opposite chiral charges.

In order to better understand the Weyl points in TaAs, we first examine the band crossings near the Fermi level in the absence of spin–orbit coupling. These band crossings take the form of a closed curve, or line node, on the *M*_*x*_ and *M*_*y*_ mirror planes of the BZ, shown in red and blue in Fig. 3a. We present the band structure near one of the red nodal lines, on the *M*_*x*_ mirror plane, in Fig. 3b. The conduction and valence bands dip into each other, giving rise to a line node crossing. Next, we include spin–orbit coupling. This causes each line node to vaporize into six Weyl points shifted slightly away from the mirror planes, shown as small circles in Fig. 3a. There are 24 Weyl points in total: 8 Weyl points on the *k*_*z*_=2*π*/*c* plane, which we call *W*_1_, and 16 Weyl points away from the *k*_*z*_=2*π*/*c* plane, which we call *W*_2_. We present the band structure near one of the Weyl points *W*_1_ in Fig. 3c, where a point band touching is clearly observed. We have calculated the momentum space locations of the Weyl nodes for the entire TaAs family. The *k*-space location for Weyl node *W*_1_ is (0.0072, 0.5173, 0), (0.0074, 0.5191, 0), (0.0025, 0.4884, 0) and (0.0028, 0.4901, 0) for TaAs, TaP, NbAs and NbP in the unit of (2*π*/*a*, 2*π*/*a*, 2*π*/*c*), respectively. The *k*-space location for Weyl node *W*_2_ is (0.0185, 0.2831, 0.6000), (0.0156, 0.2743, 0.5958), (0.0062, 0.2800, 0.5816) and (0.0049, 0.2703, 0.5750) for TaAs, TaP, NbAs and NbP in the same unit, respectively.

Next, we consider how the Weyl points project on the (001) surface BZ. We show a small region in the surface BZ around the 
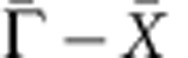
 line, which includes the projections of six Weyl points, two from *W*_1_ and four from *W*_2_. A schematic of the projections of all 24 Weyl points on the surface BZ is shown in Fig. 3e. We find that for all points *W*_2_, two Weyl points project on the same point in the surface BZ. We indicate the number of Weyl points that project on a given point in the surface BZ in Fig. 3e. Lastly, we study the chirality of the Weyl points by calculating the Berry flux (gauge field) through a closed surface enclosing a Weyl point. We call positive the chiral charges that are a source of Berry flux and negative the chiral charges that are a sink of Berry flux. We colour the positive chiral charges black and the negative chiral charges white. In Fig. 3f, we show the spin texture of the band structure near two Weyl points *W*_1_. In this case, the spin texture presents a source–sink flow around Weyl points. Moreover, it turns out that the points *W*_2_ project on the surface BZ in pairs that carry the same chiral charge.

### Topological Fermi arc surface states

**Figure 4:**
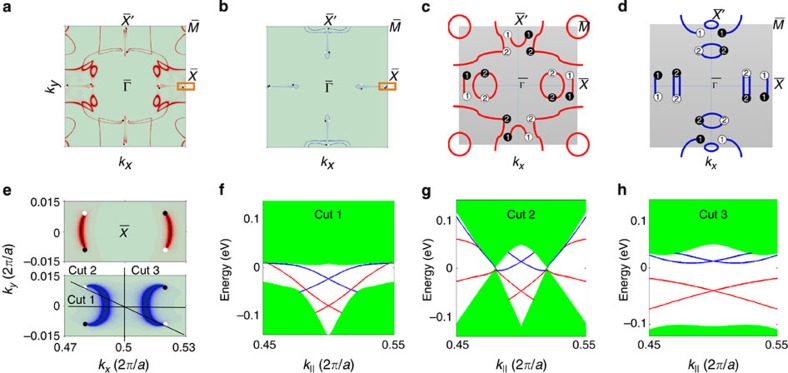
Topological Fermi arc surface states in TaAs. (**a**) The (001) surface states on the top surface of TaAs. (**b**) The same as panel **a** but on the bottom surface. The black and white circles denote the Weyl points, the shaded regions represent the spectral weight of some additional bulk bands near the surface region, whereas the sharp, red or blue curves show the surface states. (**c**,**d**) Schematics of the surface Fermi surfaces for the top and the bottom surfaces. (**e**) A close-up of the band structure on both the top and the bottom surfaces near the 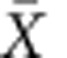 point, as indicated by the orange squares in panels **a** and **b**. (**f**–**h**) Energy dispersions of the electronic structure along three momentum space cuts, as noted in panel **e**.

Another key signature of a Weyl semimetal is the presence of Fermi arc surface states that connect the Weyl points in pairs in the surface BZ. We present calculations of the (001) surface states in Fig. 4. We show the surface states on the top surface in Fig. 4a and the bottom surface in Fig. 4b. The black and white circles denote the Weyl points, the shaded regions represent the spectral weight of some additional bulk bands near the surface region, whereas the sharp red or blue curves show the surface states. We find surface Fermi arcs that connect Weyl points of opposite chirality in pairs. To better understand the rich structure of the Fermi arcs, we show a schematic of the surface states on the top surface in Fig. 4c and the bottom surface in Fig. 4d. We note that Fig. 4c,d are only designed to show the connectivity of the Fermi arcs and the Weyl nodes. The detailed shape of the Fermi arcs does not necessarily match up with our calculation results in Fig. 4a,b. Note that one Fermi arc connects each pair of points *W*_1_. However, two Fermi arcs connect to each projection of points *W*_2_, because they project in pairs with the same chiral charge, as discussed above. This leads to Fermi arcs that connect the points *W*_2_ in a closed loop of surface states. The largest Fermi arc loop on the top surface threads through four projected Weyl points in the surface BZ. We also note that the Fermi surface of the surface states from the top is very different from that of the bottom (Fig. 4a,b), consistent with broken inversion symmetry in this system. In addition, because the presence of a surface breaks the *C*_4_ screw symmetry, the surface states are also very different along the *k*_*x*_ and *k*_*y*_ directions of the surface BZ. Finally, we observe closed Fermi surfaces that do not intersect Weyl points and do not form Fermi arcs. These extra Fermi surfaces reflect how different ways of annihilating the Weyl points would give rise to an insulator with a different topological invariant (see the Discussion below). We present a particularly simple set of Fermi arcs arising near the 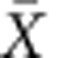 point in Fig. 4e, including surface states from both the top and bottom surfaces. To visualize the arc nature of the surface states, we present three energy dispersion cuts along the directions indicated in Fig. 4e. Along Cut 1, shown in Fig. 4f, we see a Dirac cone connecting the bulk valence and conduction bands across the bulk bandgap, exactly like a topological insulator. Along Cut 2, shown in Fig. 4g, we see the projected bulk Weyl cones, with surface states which pass through the Weyl points. Lastly, in Cut 3, shown in Fig. 4h, we observe a full bandgap. The surface states along this cut are trivial because they do not connect across the bulk bandgap.

## Discussion

A number of candidates for a Weyl semimetal have been previously proposed. However, existing proposals have proven difficult to carry out because they rely on magnetic ordering to break time-reversal symmetry or fine-tuning of the chemical composition of an alloy. Magnetic order can be difficult to predict from first-principles, may be difficult to measure experimentally, and most importantly, may not form large enough domains in a real sample for the properties of the Weyl semimetal to be preserved. Fine-tuning chemical composition is typically challenging to achieve and introduces disorder, limiting the quality of single crystals. For instance, a proposal for a Weyl semimetal in Y_2_Ir_2_O_7_^6^ assumes an all-in, all-out magnetic order, which is challenging to verify in experiment^29^,^30^. Another proposed Weyl semimetal, HgCr_2_Se_4_ (ref. 25), has a clear ferromagnetic order, but because of the cubic structure there is no preferred magnetization axis, likely leading to the formation of many small ferromagnetic domains. Moreover, a proposal in Hg_1−*x*−*y*_Cd_*x*_Mn_*y*_Te^26^ requires straining the sample to break cubic symmetry, applying an external magnetic field and fine-tuning the chemical composition. Another proposal for the inversion breaking compounds LaBi_1−*x*_Sb_*x*_Te_3_ and LaBi_1−*x*_Sb_*x*_Te_3_ (ref. 27) requires fine-tuning the chemical composition to within 5%. Despite extensive experimental effort, a Weyl semimetal has not been realized in any of these compounds. We believe that in large part the difficulty stems from relying on magnetic order to break time-reversal symmetry and fine-tuning the chemical composition to achieve the desired band structure. We note that, in contrast to time-reversal symmetry-breaking systems, large single crystals of inversion symmetry-breaking compounds exist, such as the large bulk Rashba material BiTeI, where the crystal domains are sufficiently large so that the Rashba band structure is clearly observed in photoemission spectroscopy^31^. We propose that TaAs overcomes the difficulties of previous candidates because it realizes a Weyl semimetal in a stoichiometric, inversion-breaking, single-crystal material without compositional modulations.

**Figure 5:**
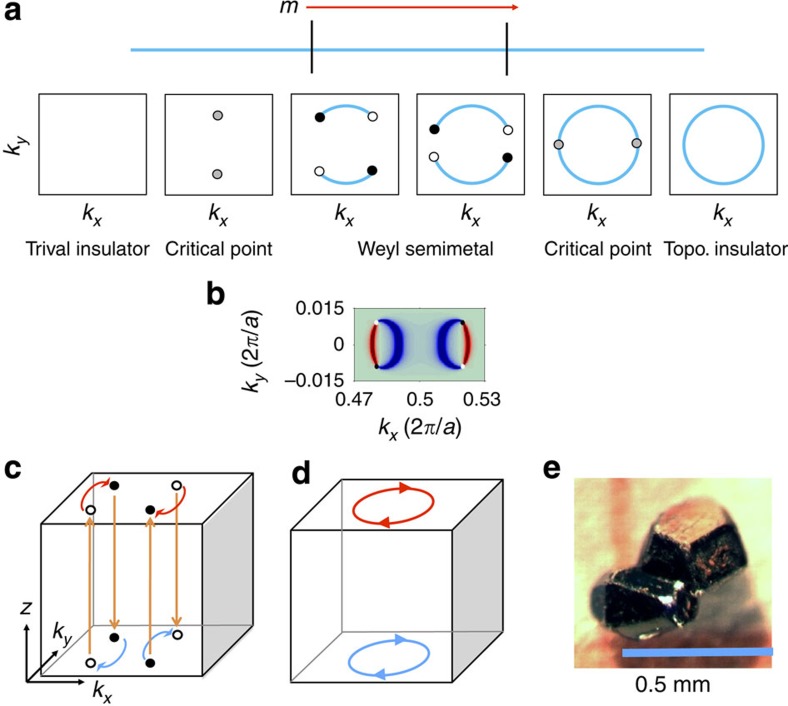
Topological phase transitions of an inversion breaking Weyl semimetal. (**a**) A Weyl semimetal can be understood as an intermediate phase between a trivial insulator and a topological insulator as a function of a tuning parameter *m*. The grey circles represent the band touchings at the critical point, each of which is composed of two degenerate Weyl nodes. The black and white circles are the Weyl nodes with positive and negative chiral charges. The blue lines are the topological surface states. (**b**) The calculated Fermi surface of TaAs near the 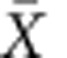 point. (**c**) In the Weyl semimetal TaAs, electrons exhibit an unusual path in real and momentum (*z*−*k*_*x*_−*k*_*y*_) space under an external magnetic field along the *z* direction. The orange arrows show the real-space motion of the electrons between the top and the bottom surfaces. The blue and red arrows show the electron's momentum space trajectories tracing out the constant energy contour of the Fermi arcs on the surfaces. (**d**) In the topological insulator Bi_2_Se_3_, an electron tracing out a surface state constant energy contour will not encounter a bulk state and the wavefunction will always remain localized on the same surface. (**e**) An image of TaAs single crystals we have grown.

Next, we provide some general comments on the nature of the phase transition between a trivial insulator, a Weyl semimetal and a topological insulator, illustrated in Fig. 5a. When a system with inversion symmetry undergoes a topological phase transition between a trivial insulator and a topological insulator, the bandgap necessarily closes at a Kramers' point. If we imagine moving the system through the phase transition by tuning a parameter *m*, then there will be a critical point where the system is gapless. In a system which breaks inversion symmetry, there will instead be a finite range of *m* where the system remains gapless, giving rise to a Weyl semimetal phase. In this way, the Weyl semimetal phase can be viewed as an intermediate phase between a trivial insulator and a topological insulator, where the bulk bandgap of a trivial insulator closes and Weyl points of opposite chiral charge nucleate from the bulk band touchings. As *m* is varied, the Weyl points thread surface states through the surface BZ and eventually annihilate each other, allowing the bulk bandgap to reopen with a complete set of surface states, giving rise to a topological insulator. This understanding of a Weyl semimetal as an intermediate phase between a trivial insulator and a topological insulator offers some insight into the closed Fermi surfaces we find in TaAs around the 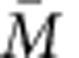 point of the top surface and the 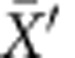 point of the bottom surface. We propose that these surface states reflect the topological invariant we would find if we annihilated the Weyl points to produce a bulk insulator. We consider the bottom surface, and annihilate the Weyl points in pairs in the obvious way to remove all surface states along 
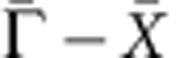
. Then, we can annihilate the remaining Weyl points to produce two concentric Fermi surfaces around the 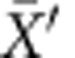 point. Since this is an even number of surface states, we find that this way of annihilating the Weyl points gives rise to a trivial insulator. If, instead, we annihilate the remaining Weyl points to remove the Fermi arc connecting them, only the closed Fermi surface would be left around 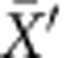, giving rise to a topological insulator. A similar analysis applies to the top surface.

Finally, we highlight an interesting phenomenon regarding how an electron travels along a constant energy contour in our predicted Weyl semimetal, TaAs, under an external magnetic field perpendicular to the surface. Figure 5b shows the calculated Fermi surface contours on both the top and the bottom surfaces near the 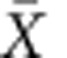 point. We consider an electron initially occupying a state in one of the red Fermi arcs, whose wavefunction is therefore localized on the top surface in real space. We ask how the wavefunction evolves as the electron traces out the constant energy contour. As the electron moves along the Fermi arc (the red arc on the left-hand side of Fig. 5c), it will eventually reach a Weyl point, where its wavefunction will unravel into the bulk. The electron will then move in real space to the bottom surface of the sample. Then it will follow a Fermi arc on that surface (the blue arc on the left-hand side of Fig. 5b), and reach another Weyl point. The electron will next travel through the bulk again and reach the top surface, returning to the same Fermi arc where it began. This novel trajectory is predicted to show exotic surface transport behaviour as proposed in ref. 23. This is just one example of the unique transport phenomena expected in Weyl semimetals. We hope (Fig. 4e) that our theoretical prediction will soon lead to the experimental discovery of the first Weyl semimetal in nature. In fact, during the review of this article, evidence for the experimental discovery of Weyl semimetal states in TaAs and NbAs have emerged 32,33,34,35, as we have predicted here.

## Methods

First-principles calculations were performed by OpenMX code based on norm-conserving pseudopotentials generated with multi-reference energies and optimized pseudoatomic basis functions within the framework of the generalized gradient approximation of density functional theory (DFT)^36^,^37^,^38^. Spin–orbit coupling was incorporated through *j*-dependent pseudo-potentials^39^. For each Ta atom, three, two, two and one optimized radial functions were allocated for the *s*, *p*, *d* and *f* orbitals (*s*3*p*2*d*2*f*2), respectively, with a cutoff radius of 7 Bohr. For each As atom, *s*3*p*3*d*3*f*2 was adopted with a cutoff radius of 9 Bohr. A regular mesh of 1,000 Ry in real space was used for the numerical integrations and for the solution of the Poisson equation. A *k* point mesh of (17 × 17 × 5) for the conventional unit cell was used and experimental lattice parameters were adopted in the calculations. Symmetry-respecting Wannier functions for the As *p* and Ta *d* orbitals were constructed without performing the procedure for maximizing localization and a real-space tight-binding Hamiltonian was obtained^40^. This Wannier function-based tight-binding model was used to obtain the surface states by constructing a slab with 80-atomic-layer thickness with Ta on the top and As on the bottom.

## Additional information

**How to cite this article:** Huang, S.-M. *et al*. A Weyl semimetal with surface Fermi arcs in the transition metal monopnictide TaAs class. *Nat. Commun*. 6:7373 doi: 10.1038/ncomms8373 (2015).
